# Biological Activity of Propolis-Honey Balm in the Treatment of Experimentally-Evoked Burn Wounds

**DOI:** 10.3390/molecules181114397

**Published:** 2013-11-21

**Authors:** Żaneta Jastrzębska-Stojko, Rafał Stojko, Anna Rzepecka-Stojko, Agata Kabała-Dzik, Jerzy Stojko

**Affiliations:** 1 Department of Anesthesiology and Intensive Care School of Medicine, Medical University of Silesia in Katowice, Medyków 14, 40-752 Katowice, Poland; E-Mail: zak@czkstojko.pl; 2 Department of Women Health, School of Health Sciences, Medical University of Silesia in Katowice, Medyków 12, 40-752 Katowice, Poland; 3 Department of Pharmaceutical Chemistry, School of Pharmacy and Laboratory Medicine, Medical University of Silesia in Katowice, Jagiellońska 4, 41-200 Sosnowiec, Poland; E-Mail: annastojko@sum.edu.pl; 4 Deparment of Pathology School of Pharmacy and Laboratory Medicine, Medical University of Silesia in Katowice, Ostrogórska 3, 41-200 Sosnowiec, Poland; E-Mail: adzik@sum.edu.pl; 5 Department of Hygiene, Bioanalysis and Environmental Studies School of Pharmacy and Laboratory Medicine, Medical University of Silesia in Katowice, Kasztanowa 3A, 41-200 Sosnowiec, Poland; E-Mail: jstojko@sum.edu.pl

**Keywords:** propolis, bee honey, burn wound, collagen, hydroxyproline

## Abstract

Medicines of biogenic origin with micro-organic, regenerative and analgesic properties are becoming more and more significant in the treatment of burn wounds. These properties are found in apitherapeutics such as propolis and honey—products collected and processed by a honey bee. Their effect on the course of the healing processes is multidirectional. The aim of the study was a histopathological and biochemical analysis of the processes of scar formation in experimentally evoked burn wounds in white pigs treated with the 1% and 3% Sepropol balms containing standardized extracts of propolis and honey. The results were compared with the therapeutic effects obtained with dermazin cream (1% silver sulfadiazine). The level of collagen was determined in the wounds treated with 1% and 3% Sepropol and compared with the collagen level in healthy skin and wounds treated with dermazin. Granulation and regenerated epithelium formation times were compared, with the 3% Sepropol being by far the most effective. The 3% Sepropol also increased the collagen level to 116% with the control sub-groups scoring between 80% and 98%. The results show the healing process of burn wounds in pigs treated with the Sepropol balm starts earlier and has a faster course than the standard dermazin therapy.

## 1. Introduction

The treatment of burn wounds is a significant problem of clinical medicine. Back in the early 20th century the local treatment of burn wounds was limited to the application of antiseptics and their anti-bacterial properties. Microorganisms find extremely favorable conditions for intensive development in a burn wound and the damage to the skin, which is a natural protective barrier, leads to protein denaturation, necrosis and exudate, with an ischemic zone surrounded by edema. As a consequence, the defensive mechanisms, both humoral and cellular, are impaired. Proper assessment of the burn depth determines the decision regarding the local treatment—either conservative or surgical [1].

In the light of the latest scientific reports, medicines of biogenic origin with their anti-microorganic, regenerative and analgesic properties are becoming more and more significant in the treatment of burn wounds [2]. These properties are found in apitherapeutics such as propolis and honey products collected and processed by a honey bee. Standardized extracts obtained from bee products constitute the base for various apipharmaceutics and the standardized propolis extract is becoming more widely used in burn wounds treatments [3,4,5].

Propolis is one of the most significant bee products. It contains substances with high biotic activities which are especially valuable in the stimulation of reconstructive processes of damaged tissues. The activity of the standardized propolis extracts refers both to the repair and regeneration processes. Propolis stimulates the vascular endothelial growth factor and significantly intensifies cellular proliferation confirmed by the growth of H3 histone [6].

Propolis is a resinous substance collected by bees from various tree species and enriched with the secretion of their fauces glands. It consists of approximately 300 synergistic compounds, with the most important roles being played by phenolic compounds and their anti-oxidizing, anti-rheumatic and disinfectant properties; flavonoids and their anti-inflammatory, anti-microorganism, and anti-neoplastic properties; terpenes with their antibiotic and immune-stimulating properties; and lipid-wax substances which decrease the LDL fraction of cholesterol in blood. Other important elements present in propolis include calcium, manganese, magnesium, zinc, copper, silicon and iron. Propolis is also rich in pro-vitamin A (β-carotene), vitamin A (retinol), vitamins B1, B2, B5, B6, C, E, D and also proteins and carbohydrates. Numerous scientific studies have proven various and multidirectional biotic properties of both propolis and also other bee products [7] such as antibacterial, anti-mycotic, antiviral, anti-protozoan, anti-oxidizing, anti-inflammatory, anesthetizing, hypotensive, anti-aggregation, regenerative, cholepoietic, anti-neoplastic and immune-modulating [8,9,10]. Some authors refer to propolis as to the antibiotic of the 21st century because of its synergic activity with other antibiotics such as oxytetracyclines, gentamycins and bacitracins. The antibacterial activity of propolis results from the fact that it contains flavonoids and phenolic acid esters [11,12]. Propolis successfully fights *Staphylococcus aureus* which is resistant to methicillin, streptococci, *Moraxella catarrhalis* and some strains of tuberculosis mycobacteria. It counteracts the development of *Candida*, *Sporothrix* and *Paracoccidoides* fungi and suppresses the multiplication of influenza and herpes viruses which are the most frequent viral etiological factors in oral and nasal cavity, lungs or cerebrospinal meninges inflammations, and also destroys protozoon causing trichomoniasis, toxoplasmosis and lambliasis [13,14].

Honey and standardized propolis extract balm have been found most effective in the treatment of burn wounds since when used together, these two substances show all the necessary burn wound treatment functions: antibacterial, reparative and anesthetic [15]. They not only accelerate the granulation creation but also facilitate scar formation.

Honey consists of water, sugars, organic acids, nitrogen compounds, bio-elements, flavonoids, carotenoids, ethereal oils, phospholipids, vitamins and microelements. Its carbohydrates include mainly monosaccharides (glucose, fructose and slight amounts of polysaccharides: sucrose and maltose) while its protein compounds are mainly enzymes, simple proteins—albumins and globulins and free amino acids originating from bee fauces glands. They condition the pharmacological activity [16].

The only variety of honey used in the treatment of burn wounds is the one produced from the nectar of entomophilous plants [17,18,19,20]. The best known pharmacological property of honey is its antibacterial activity [21,22] which results from its rich chemical composition, low pH, high concentration of carbohydrates and release of hydrogen peroxide—a by-product of glucose oxidation under the influence of glucose oxidase—the enzyme synthesized in bee fauces glands. Other anti-microorganic compounds of honey include lysozyme, apidicine, terpenes, flavonoids, organic acids and substances from oil plants nectar and honeydew of coniferous trees – eucalyptol, thymol, menthol, camphene and other [23]. Honey has a synergistic effect by intensifying the antibacterial activity of known antibiotics against multi-resistant microorganisms [24]. It reveals a significantly stronger bactericidal activity against G+ bacteria, it produces an effect on *Staphylococcus aureus* [25], streptococci: *Streptococcus pyogenes* and *Strep. Pneumonia*, but has a weaker effect on G- bacteria of the Enterobacteriacae family: *E.coli*, *Klebsiella pneumoniae*, *Proteus vulgaris*, *Salmonella*, *Shigella* or non-fermenting rods of Pseudomonas family [26,27]. Additionally, honey is a valuable energetic product [28,29] with its properties such as anti-oxidation [30], detoxication, immune-modulating, anti-allergic, regenerating and tranquilizing [31,32]. 1% honey concentration stimulates monocytes to release cytokines IL-1 and IL-6 and TNF-α which activate the immunological response [33]. The above-listed honey properties allow for its wide application in various fields of medicine, e.g., in dentistry, gynecology, urology, dermatology, internal diseases [32,33,34,35,36], in the treatment of difficult to heal wounds, burns, bedsores, trophic crural ulcerations, ulceration of stomach mucosa, inflammations of upper airways, oral cavity and parodontium diseases, and especially in treating dry socket [37,38,39,40,41,42,43].

While the literature does list therapeutic activities of the standardized honey extract and propolis, there have been no publications on the effects of their simultaneous use as a mixture. It would seem that such a preparation should condition the synergistic activity and, consequently, shorten the healing time and also improve the esthetics of the emerging scar.

The main aim of the study was the histopathological and biochemical analysis of the scar formation processes in experimentally-evoked burn wounds in white pigs, treated with standardized honey extract and propolis balm, 1% and 3% Sepropol, and their comparison to the effects obtained with conventional therapeutic means—dermazin. Additionally, the collagen levels, which have an impact on the acceleration of the healing process and the quality of the emerging scar were also determined and compared.

## 2. Results and Discussion

### 2.1. Clinical Assessment

The clinical assessment of burn wounds covered their size and appearance as well as the condition of the skin around the wound. The animals were put in general anesthesia on days 1, 3, 7, 14 and 21 of the experiment and the wounds were then measured with a measuring rule to the accuracy of 1 mm. The initial wound size was considered to be 15 × 30 mm, *i.e.*, the size of the burning pad. All other parameters of the wound and its surroundings were assessed with a standard physical examination. The granulation formation process was assessed as well as the course of scar organization taking place on the wound surface.

The wounds in the D1 (1% Sepropol) and D2 (3% Sepropol) sub-groups were clearly smaller than in the K1 and K2 sub-groups as soon as day 3, with the edema and reddening around the wound also being smaller.

On day 7, the skin around the wounds was red, cracked and swollen in the control K sub-groups, while in the Sepropol sub-groups no edema or reddening were observed (yet the serous exudate was still present).

On day 14, the wounds were tightly covered with crust; in control sub-groups the exudate remained, while in the studied sub-groups red epithelium emerged at the wound edges, where crust was falling off from the wound surface.

On day 21, the wounds size remained unchanged in the control group, with the crust falling off from the surface and a dark pink scar being visible under the crust. In the studied sub-group D1 (1% Sepropol), the wounds were fully covered with light pink epithelium, while in the sub-group D2 (3% Sepropol) the wounds were healed and covered with an organized scar.

The wound healing process was significantly different among the various sub-groups. In K1, the wounds were dry and cracked and they continued to crack and bleed even after crust formation. In K2, the wounds were dry and cracked and there was edema and a zone of hyperemia around them. The healing process started on day 14 but the wound size did not decrease. On day 21, the crust started to fall off from wound surfaces and a scar started to form underneath. In both Sepropol sub-groups the wounds were wet and the reddening around the wound was less extensive. On day 14 the wounds got visibly smaller and the crust was falling off at the edges, which was especially clearly visible for the 3% Sepropol D2 sub-group. On day 21, the 1% Sepropol sub-group’s wounds got smaller and their surface was covered with a delicate epithelium. However, in the sub-group D2 (3% Sepropol) the wounds were covered with an organized scar and could be considered healed.

### 2.2. Histopathological Test Results

**Figure 1:**
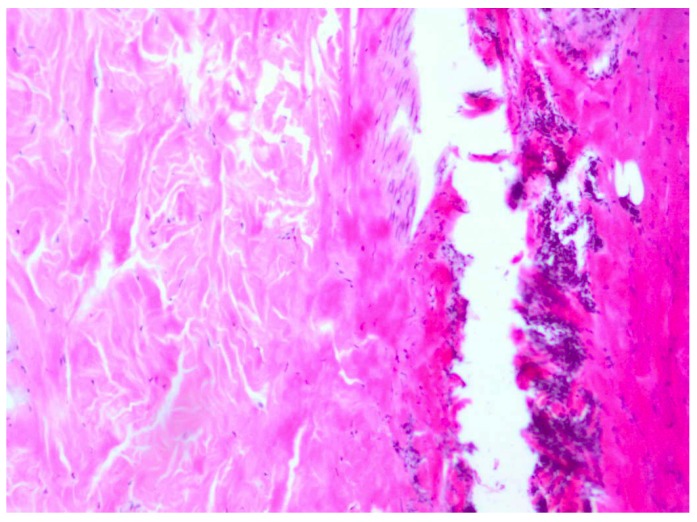
Day 7, K1-0.9% NaCl, fibrinous exudate, stratified squamous epithelium defect, visible necrotic lesions, coagulative necrosis and inflammatory infiltration (enlargement 150×, HE).

**Figure 2:**
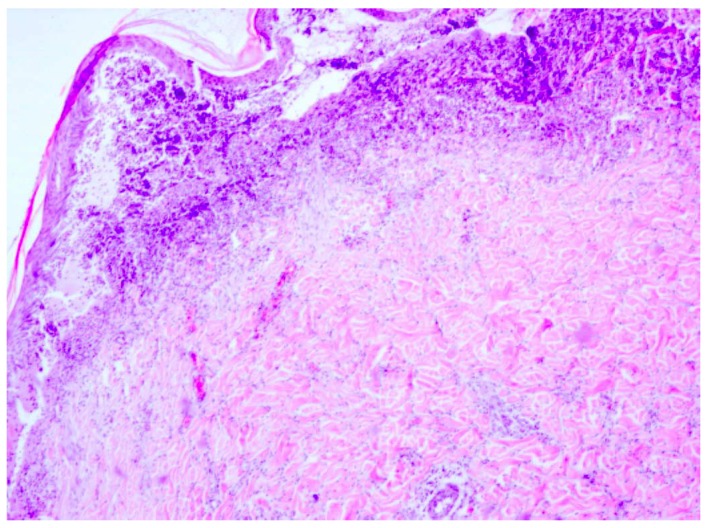
Day 7, K2-dermazin an extensive, deep coagulative necrosis, wound covered with fibrin masses and extensive inflammatory infiltration, fibrin presence (enlargement 150×, HE).

**Figure 3:**
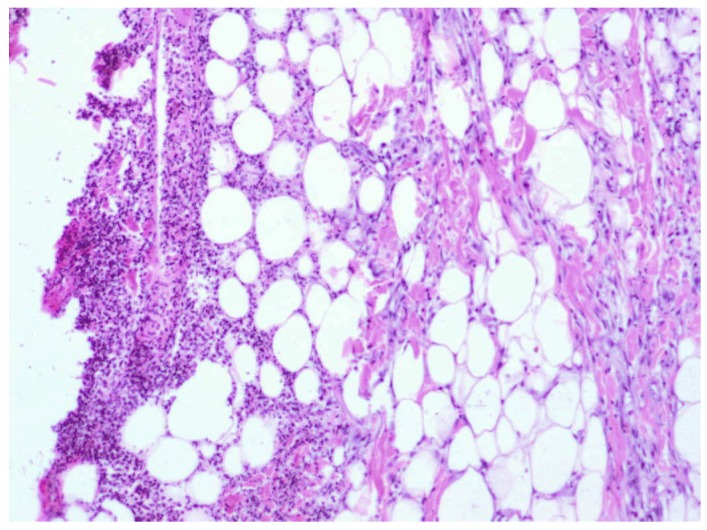
Day 7, D1-1% Sepropol-epithelium defect with necrosis, inflammatory infiltration with granulocytes (enlargement 150×, HE).

**Figure 4:**
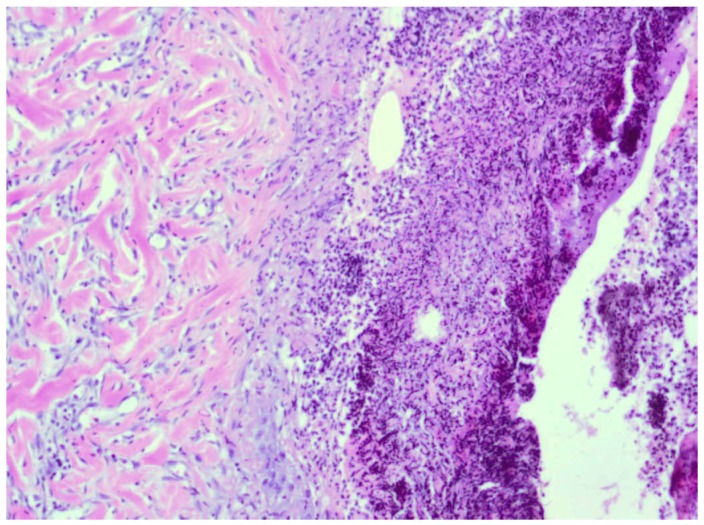
Day 7, D2-3% Sepropol, inflammatory exudate with great amount of granulocytes, on the bottom of the wound and at its edges granulation process marked (enlargement 150×, HE).

**Figure 5:**
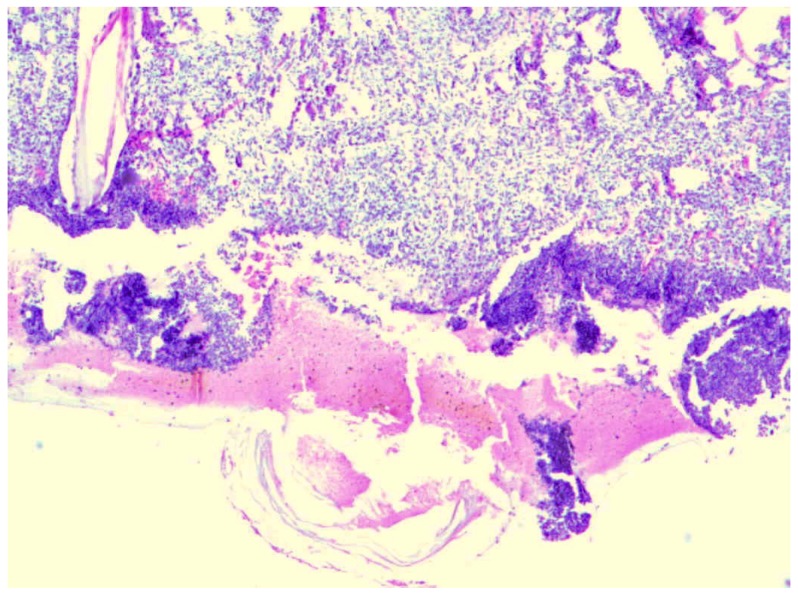
Day 21, K1-0.9% NaCl, defect visibly filled in with granulation tissue and crust covering the wound, inflammatory infiltration reach granulocytes within the skin damage borders (enlargement 150×, HE).

**Figure 6:**
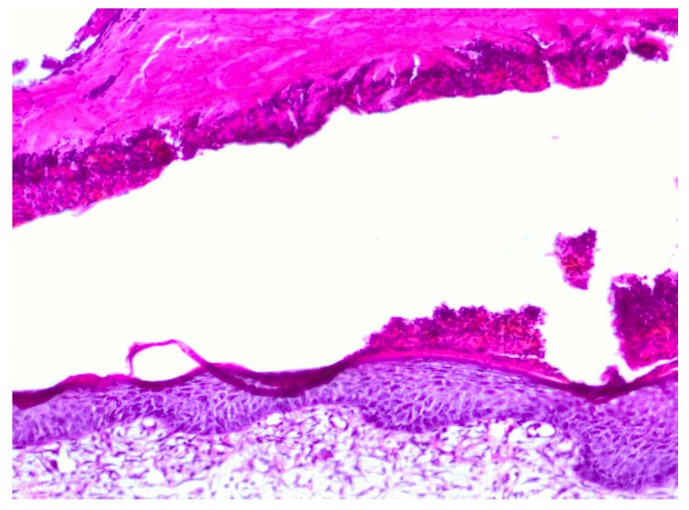
Day 21, K2-dermazin, crust visible at wound edges visible, fresh granulation tissue underneath, inflammatory infiltration, visible unobstructed blood vessels (enlargement 150×, HE).

**Figure 7:**
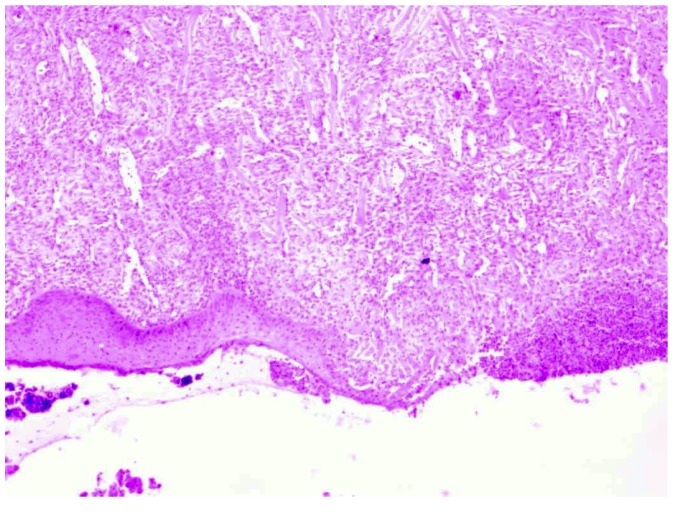
Day 21, D1-1% Sepropol, the entire defect covered with regenerated epidermis, defect filled with granulation with predominant collagen fibers (enlargement 150×, HE).

**Figure 8:**
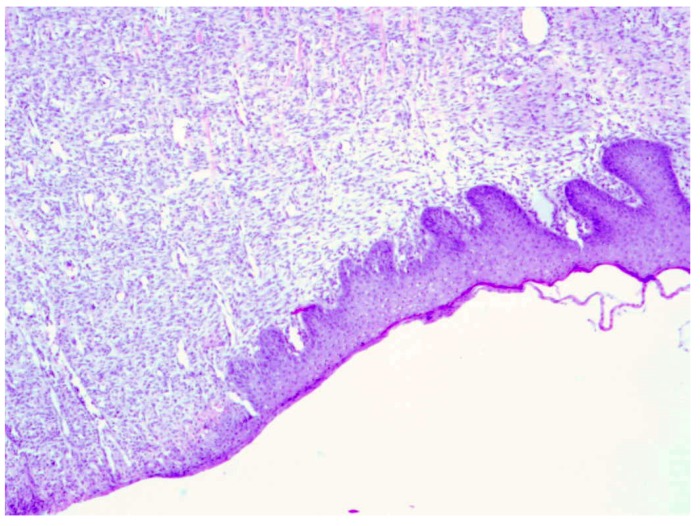
Day 21, D2-3% Sepropol, healed burn wound, granulation tissue with predominant collagen fibers building up connective tissue scar (enlargement 150×, HE).

We analyzed the wound cleansing, ground substance creation, proliferation of blood vessels and collagen fibers formation. All these elements appear in the successive stages of granulation and scar formation. On day 3, the histopathological picture in all sub-groups was similar, fibrous exudate could be observed on the wound surfaces, as well as stratified squamous epithelium defect and deep necrotic lesions reaching fatty tissue. An inflammatory infiltration could be observed around blood vessels. On day 7 the histopathological picture changed significantly especially in the 3% Sepropol sub-group. Figure 1, Figure 2, Figure 3, Figure 4, Figure 5, Figure 6, Figure 7 and Figure 8 show some of these effects.

On day 14 the histopathological picture in each sub-group was significantly different. In the control sub-groups the epithelium defect could still be observed, also present were the coagulative necrosis and inflammatory infiltration and the first forms of granulation tissue appeared as well. In the studied sub-groups the healing process was much more visible and the epithelium defect started to fill in with fresh granulation tissue. On day 21 the improvement was even more significantly visible in the Sepropol sub-groups *vs.* the K sub-groups.

Histopathological test results show considerable discrepancies in the healing process of burn wounds depending on the applied preparation in favor of experimental sub-groups D1 and D2. The pathological process in each group was identical, however, the repair processes started at different times. In wounds washed out with isotonic salt solution, stratified squamous epithelium defects as well as masses of coagulative necrosis were observed throughout the whole experiment. On the last days of the experiment, the defects were filled with granulation, however, an inflammatory exudate and tissue debris were observed on the wound surface.

In the dermazin group, on the 3rd and 7th day of the experiment, the wounds picture was similar to the group dressed with isotonic salt solution. On the 14th day, the first signs of granulation were observed, however, necrotic masses with epithelium defects and inflammatory infiltration were still present. On the 21st day, the granulation process was visible, yet the epithelium defects and inflammatory process remained.

In the 1% Sepropol sub-group, the differences were significant on day 14. The epithelium defects were filled with granulation, while on day 21 the granulation was covered with regenerated epithelium. In the 3% Sepropol sub-group, the first signs of granulation, both on the wound surface and on its bottom, were visible on day 7. Inflammatory infiltration with a considerable amount of granulocytes was observed in the preparation, which proves the wound was being cleaned and prepared for epithelium regeneration. On day 14, the regenerating epithelium could be observed, with mature granulation underneath. On day 21, the defects were filled with granulation with a considerable amount of collagen fibers building up the connective-tissue scar. The experiments thus proved that the best effect was obtained with the application of 3% Sepropol.

### 2.3. Biochemical Test Results

**Figure 9:**
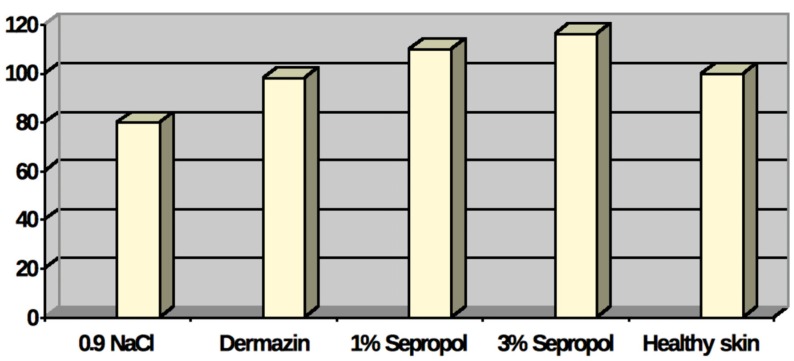
Proportional level of collagen in burn wounds scars on the 28th day of the experiment as compared to healthy skin. Collagen level in healthy skin is assumed to equal 100%.

Biochemical tests supplemented the histopathological assessment. The collagen level in burn wound scars was determined and compared with healthy skin parameters. This was done with the indirect method by determining level of hydroxyproline—the collagen-specific amino acid, which, practically speaking, does not exist in other organic proteins. The quantitative determination of hydroxyproline is used for the assessment of the stage of repair processes. Assuming that the collagen level in healthy skin is a constant, the results (Table 1 and Figure 9) show a clearly advantageous effect of 1% and 3% Sepropol on the full reconstruction of collagen structure in a newly-emerged scar. The similar cannot be said of dermazin—in this case the collagen level was under the standard level for healthy skin.

**Table 1:** Hydroxyproline and collagen levels in μg/mg of dry mass in burn wounds scars on the 28th day of the experiment as compared to healthy skin.

	Healthy skin	Burn wounds			
		0.9% NaCl	Dermazin	1% Sepropol	3% Sepropol
hydroxyproline [g/mg dry mass ± SD]	58.2 [±0.73]	45.5 [±0.67]	57.5 [±0.81]	64.2 [±0.47]	68.4 [±0.63]
collagen [g/mg dry mass]	464	360	456	512	544

### 2.4. Statistical Analysis

In order to verify the hypothesis regarding the effect of burn wound treatment method on the average hydroxyproline level expressed in μg/mg of dry mass in burn wound scars on day 28, a one-way analysis of variance for independent samples was carried out (Table 2).

A statistically significant effect of burn wound treatment method was obtained: NaCl, dermazin, 1% Sepropol or 3% Sepropol F (4.32) = 101.6757, *p <* 0.000001. Analysis results confirm all the predictions regarding mean values. The hypothesis regarding the equality of mean values at the level of *p <* 0.000001 was rejected.

**Table 2:** One-way analysis of variance for independent samples.

Variable	Variance analysis (Determined effects are significant with *p* < 0.0500)							
	SS effect	df effect	MS effect	SS error	df error	MS error	F	p
Hydroxyproline level	2745.234	4	686.311	216.000	32	6.750	101.676	0.0000

SS effect—sums of squares among groups; df effect—number of degrees of freedom among groups; MS effect—mean square among groups; SS error—sums of squares inside groups (remainders); df error—number of degrees of freedom inside groups (remainders); MS error—mean square inside groups; F—Fisher test value; p**—**probability level *p*.

In order to confirm which burn wound treatment methods stand out of the control group values the most and which differ among themselves most significantly, the test of differences among mean values from each group was carried out. *Post-hoc* comparisons with the LSD test showed that only the dermazin therapy does not differ statistically significantly from the results obtained for healthy skin. For the studied preparation, regardless of the active substance concentration, statistically significant differences were shown (Table 3).

**Table 3:** *p* values showing significance levels for subsequent mean pairs.

Method	LSD test; Variable (Determined differences are significant with *p* < 0.05000)				
	{1} M = 45	{2} M = 57	{3} M = 64	{4} M = 68	{5} M = 58
NaCl 0.9% {1}		0.000000	0.000000	0.000000	0.000041
Dermazin {2}	0.000000		0.000002	0.000000	0.717403
Sepropol 1% {3}	0.000000	0.000002		0,002603	0.035857
Sepropol 3% {4}	0.000000	0.000000	0.002603		0.000922
Healthy skin {5}	0.000041	0.717403	0.035857	0.000922	

Medicines containing standardized, pharmacologically active compounds of biogenic origin, e.g., apitherapeutics, are becoming more and more popular burn wounds therapies as they have a positive effect on the wound healing process by stimulating the cellular cycle and synthesis of extracellular matrix components. The scientific interest in and meticulous studies of apitherapeutics revealed their therapeutic effectiveness in various medical disorders. However, there is always the question of how to select the optimal concentration of the therapeutic substance in order to fully expose its therapeutic effects and simultaneously minimize side-effects.

Various authors have confirmed the effectiveness of apitherapeutics and their advantages over other commonly applied pharmaceutics. In 2002, Gregory *et al*. carried out a prospective study comparing the course, efficacy and outcome of the treatment of 2nd grade burn wounds dressed with propolis skin cream and silver sulfadiazine [44]. The study covered middle-aged patients with bilateral burns which did not exceed 20% of the body. One part of the wounds was dressed with the propolis skin cream and the second part with silver sulfadiazine salt. The assessment of the course of treatment, the inflammatory infiltration intensification, the granulation rate and the quality of the emerging scar showed that propolis was the substance of considerably better therapeutic outcome as compared to the silver sulfadiazine salt. Our own study revealed comparable therapeutic outcomes, with the 3% Sepropol balm being most effective.

Similar results were obtained by Pessolato *et al.* in 2011 [45]. The study was carried out on rats and it compared the outcomes of 2nd grade burns treatment with 5% propolis ointment and autologous amnion. Macro- and microscopic assessment of epidermis and dermis was performed, as well as morphometric assessment of the stimulation degree of collagen fibers formation. Efficacy of both applied treatment methods was comparable. Both the propolis ointment and the amnion reduced the local inflammatory infiltration. The macroscopic assessment was more favorable for propolis on the 7th day. On the following days, a comparable efficacy of both studied substances was observed in the scope of accelerating the regeneration process of damaged tissue and stimulation of collagen fibers production. As in the case of our own studies, the applied treatment methods allowed for reduction of the emerging scar, a faster come-back to normal functioning and improvement of burned patients’ lives quality. The Pessolato *et al.* study confirmed the histopathological tests results obtained in our own material. Emerging fibers of connective tissue on the 14th day of the experiment together with a simultaneous retreat of micro-capillary tubes are the evidence of a considerable acceleration of healing processes observed at the cellular level.

A frequent complication of the burn wound healing process is a bacterial infection as it considerably prolongs the therapy and often requires a modification of the treatment. In 2012, Berretta *et al.* carried out a study whose aim was to determine the most optimal standard concentration of propolis extract (EPP- AF) which gives the best outcomes in managing local skin damages in rats [46]. In the process of multiple dilutions, specific concentrations of the studied EPP-AF agent were obtained, which were later tested *in vitro* and *in vivo* against the micro-organisms most frequently present in wounds, such as: *Pseudomonas aeruginosa*, *Klepsiella pneumoniae*, *E. coli*, *Staphylococcus aureus* and *Staphylococcus epidermidis*. The best antibacterial properties and at the same time the best therapeutic outcome was obtained with the samples containing 3.6% propolis.

Propolis also reveals an advantageous effect in sun burns as was shown by Cole *et al*. in a study on hairless mice [47]. They showed that the ethanol propolis extract is a very effective UV-radiation blocker, completely eliminating the possibility of burn wounds on hairless skin. The authors demonstrated the inhibition of lipid peroxidation process with a subsequent decrease of interleukin 10 over-expression and drop of interleukin 12 level, brilliantly explaining the photo-protective activity of propolis. Interestingly, the active substance concentrations ranged between 0.0001% and 0.01% of the total solution mass.

The literature provides various study results of the second active component of Sepropol, *i.e.*, the standardized honey extract. Al-Waili *et al*. studied female patients with infectious complications in post-operative Caesarian section wounds [48]. Group A consisted of female patients treated with honey for 12 hours. Group B consisted of patients treated with 70% ethanol and iodine. Antibiotics were administered to both groups. Bacteria elimination in group A took place after 6 days (antibiotics were administered for 6.8 days) and in group B after 14.8 days (antibiotics were administered for 15 days). Full recovery in group A was after 10.7 days and in group B after 22 days. Scar size in group A was 3.6 mm and in group B 8 mm. The observed faster bacteria elimination and, in turn, acceleration of the healing process considerably reduces the hospitalization period and improves the cosmetic effect of the obtained scar.

Moreover, in a study by Subrahmanyam *et al.* [49] patients in the 1st group received the standardized honey extract and in the 2nd group—dermazin. In the first group 91% of wounds were aseptic after 7 days. In the second group, the same effect was observed in less than 7%. The granulation could be observed much earlier in patients treated with honey—7.4 days *vs.* 13.4 days for dermazin patients.

Based on the above results and our own findings, it is recommended that due to low costs, high effectiveness and easy application, honey should be considered as a valid alternative in burns treatment. While the contemporary medicine moves at a logarithmic speed, with the development of molecular biology, gene therapy, studies on stem cells, *etc.* sometimes it may be a good idea to go back to the good old therapeutic methods because we can easily confirm their effectiveness even at the molecular level.

## 3. Experimental

### 3.1. Reagents

Xylene, hematoxylin, eosin aqueous solution (1%), anhydrous ethyl alcohol (99.8%), acetone, NaOH, chloramine T 3-hydrate and perchloric acid (70%) from POCh (Gliwice, Poland). DPX mounting medium DPX and hydroxyproline from Fluka (Dresden, Germany). Tris-HCl (0.05 M), 4-(dimethylamino)benzaldehyde (98%) from Sigma-Aldrich (Munich, Germany).

### 3.2. Therapeutic Agents

The subjects of the study were Sepropol 1% and 3% balms consisting of the standardized propolis extract and the nectar polyfloral honey at a 1:1 ratio in a white petrolatum base. Authorized for consumption by Państwowy Zakład Higieny (National Institute of Hygiene) under certificate 11 153/94. The technological process of the apitherapeutic agent was developed in the Polish Foundation for Apitherapy in Katowice, Poland and dermazin (silver sulfadiazine) 1% cream, from Lek Pharmaceuticals (Ljubljana, Slovenia).

### 3.3. Tissue Materials

Approval for the study was obtained from the Regional Ethical Committee of the Medical University of Silesia in Katowice, Poland (resolution no. 25/2010 on 5 October 2010). The subject of the histopathological and biochemical tests were segments collected in the course of the experimental therapy of burn wounds. The experimental wound model was developed in accordance with the Hoekstra standard [50]. The white pig is often used as the biological experimental model for the study of the healing processes of various wounds because of the complete similarity of pig skin to the human skin, the only difference between the two being in the depth of particular histological layers. The experiment duration of 21 days was based on the Hoekstra procedure. The last day of the experiment is based on the complete healing of the wound in one of the sub-groups in our case, this was day 21 and the 3% Sepropol sub-group. Also, the biochemical and histopathological data collected on that day from the assessment of the remaining sub-groups revealed significant differences in the healing process when compared to the 3% Sepropol sub-group. Based on the fact that the data revealed the end of the healing process in the wound area in one of the sub-groups, this was the last day of the observations, and as a result, of the study. Observations were carried out on two white pigs, aged 15–16 weeks and with the weight of approximately 40 kg. In each of them and in deep general anesthesia with ketamine hydrochloride and sodium thiopental—18 burn wounds were produced on the surface of the back—nine on each side. The surface of each wound was 15 × 30 mm. The total area of burns did not exceed 10% of total skin surface. Out of 36 burns (n = 36), 18 wounds were in the control group K (n = 18) and the remaining 18 the experimental group D (n = 18). Within the control group K 2 sub-groups were created: K1 (n = 9) with wounds washed with the 0.9% solution of NaCl and K2 (n = 9) with wounds managed with silver sulfadiazine (dermazin). Within the experimental group D two sub-groups were also created: D1 (n = 9) with wounds treated with Sepropol 1% cream and D2 (n = 9) with wounds treated with Sepropol 3% cream. Each wound was dressed with an aseptic dressing which was changed once a day and contained a comparable amount of therapeutic agent.

### 3.4. Histopathological Test

The histopathological assessment of healing process was carried out on 3rd, 7th, 14th and 21st day of the experiment. The studied material was collected from three wounds in each sub-group: K1, K2, D1 and D2. Prior to material collection, the animals underwent general anesthesia. Segments included the middle part of wound, the edge of the lesion and the skin around the wound. The collected material was fixed in a 10% formalin solution for minimum 24 hours. After fixing, the tissues were treated with the aqueous solution of ethyl alcohol in the concentrations of 70% v/v, 80% v/v, 96% v/v respectively. The material was subsequently poured with acetone and xylene respectively. After that, tissues were placed in a xylene and paraffin mixture at a 1:1 ratio and then in liquid paraffin. After solidification, the paraffin was cut into 3–5 micron thick bands. Paraffin bands were treated with xylene, an ethyl alcohol/xylene mixture at a 1:1 ratio and aqueous solution of ethyl alcohol in the concentrations of 99.8% v/v, 96% v/v, 90% v/v, 80% v/v, 70% v/v respectively and then rinsed in distilled water.

The material prepared in this way was stained with the standard HE method, hematoxylin-eosin, which included staining with alkaline solution of hematoxylin, rinsing in distilled water, staining with acid solution of eosin and rinsing in distilled water. Later, preparations were treated with aqueous solution of ethyl alcohol in the concentrations of 70% v/v, 80% v/v, 90% v/v, 96% v/v, 99.8%, respectively, ethanol/xylene mixture at 1:1 ratio and rinsed in xylene in order to finally dehydrate and radiate the tissue. Slides were closed with cover glass by means of DPX-medium.

### 3.5. Biochemical Test

The biochemical test covered the determination of collagen level in scars of burn wounds managed with 0.9% NaCl, dermazin and 1% and 3% Sepropol and compared with healthy skin parameters. The determination of collagen amount was performed with the indirect method by the determination of hydroxyproline level. Quantitative hydroxyproline determinations in micrograms per milligrams of dry mass were performed in accordance with the Prockop and Underfriend method [51] on the 18th day of the experiment. In order to do so, material samples collected from burn wounds and the area around them were treated with 0.9% NaCl, dermazin and 1% and 3% Sepropol respectively containing 100 mg of degreased tissue each underwent homogenization and then ultrasound disintegration in 0.05 M of Tris-HCl. The hydroxyproline level was determined with the spectrophotometric method. The homogenate underwent the hydrolysis in HCl in the nitrogen atmosphere. Hydrolysate was neutralized with NaOH and oxidized with the chloramine T solution and perchloric acid. The product of hydroxyproline oxidation underwent a specific reaction with *p*-dimethylaminobenzaldehyde and the absorbency of the solution was measured with the wave-length of λ = 557 nm with the SP-830 Plus Metertech spectrometer. The hydroxyproline amount was calculated with the standard curve prepared for a standard hydroxyproline solution (5–20 mg/L; y = 0.0109x + 0.025; R^2^ = 0.989). The calculated hydroxyproline value was multiplied by a constant calculation coefficient equaling 8, because hydroxyproline constitutes approximately 1/8 of collagen mass. Obtained results were compared with the collagen level in undamaged healthy skin of animals taking part in the experiment.

## 4. Conclusions

The healing process of burn wounds treated with Sepropol is faster as compared to the standard dermazin therapy. Histopathological tests show that the process of scar formation in wounds treated with Sepropol starts considerably earlier as compared to the control group. The hydroxyproline level, and, in turn, the collagen level, increase in a statistically significant way in wounds dressed with Sepropol as compared to the control group. The reparatory activity of Sepropol is in a statistically significant way dependent on the active substance concentration (1% *vs.* 3%).
